# Molecular identification and antifungal susceptibility pattern of *Candida* species isolated from HIV infected Patients with candisiasis

**DOI:** 10.18502/cmm.5.1.533

**Published:** 2019-03

**Authors:** Sony Paul, Iyanar Kannan

**Affiliations:** 1 Department of Microbiology, Tagore Medical College and Hospital, Rathinamangalam, Chennai, India

**Keywords:** Antifungal agents, Antifungal resistance, Antifungal Susceptibility testing, *Candida* species, Candidiasis, Molecular identification

## Abstract

**Background and Purpose:**

Opportunistic fungal infections have been on a growing trend since the last two decades. Among the opportunistic fungal agents, *Candida *species*, Cryptococcus neoformans, *and* Aspergillus fumigatus* account for most of the life-threatening infections in immunocompromised individuals. Regarding this, the present study aimed to investigate the molecular identification and antifungal susceptibility pattern of *Candida *species isolated from HIV-infected patients*.*

**Materials and Methods:**

This study was conducted on 80 clinical samples collected from HIV-infected patients with suspected candidiasis referring to Tagore Medical College and Hospital, Rathinamangalam and Government Hospital of Thoracic Medicine, in Chennai, India, for 18 months (i.e., May 2016-December 2017). Phenotypic and molecular identification was accomplished using internal transcribed spacer region 1 (ITS1) and ITS4 primers. The antifungal susceptibility pattern of the isolates against four antifungal agents was also determined by both disk diffusion and broth dilution methods.

**Results:**

In the present study, the prevalence of candidiasis was obtained as 75% (n=60). *Candida tropicalis *was the predominant identified species. All the emerging species (i.e., *Kodamaea ohmeri, Hanseniaspora opuntiae, *and* C. orthopsilosis*) were identified through molecular identification since the phenotypic identification was inconclusive. In terms of the susceptibility pattern, 63.3% and 18.3% of the isolates were resistant to fluconazole and voriconazole, respectively. *Candida albicans *was also found to be resistant to amphotericin B.

**Conclusion:**

Molecular assay led to the identification of *K. ohmeri, H. opuntiae, *and* C. orthopsilosis*, which were multidrug-resistant. This study highlighted the need for the prompt and timely identification of clinical yeast isolates given the emergence of many rare species and their capability of causing life-threatening infections and outbreaks. In the laboratories where molecular diagnostic methods are not available, alternative services of reference laboratories can be utilized as cost-effective measures. With regard to the growing prevalence of antifungal drug resistance, antifungal susceptibility testing should be made mandatory for effective patient management.

## Introduction

The most common opportunistic fungal infection in AIDS patients is candidiasis. Opportunistic fungal infections have been on a growing trend since the last two decades. Candidiasis occurs in various clinical entities from mucocutaneous to invasive forms. *Candida *infections occur when the CD4 count is between 200-500 cells/μl, which can be the first indication of immunodeficiency. Among the opportunistic fungal agents, *C. albicans, Cryptococcus neoformans, *and* Aspergillus fumigatus* account for most of the life-threatening infections in immunocompromised individuals [1-4]. Although *C. albicans *has been considered as the most commonly isolated organism, some studies have shown that non-*albicans* *Candida *(NAC) species, including *C. tropicalis, C. krusei, *and *C. glabrata, *significantly account for infections [5-8]. These non-*albicans Candida *species are usually less susceptible to the more commonly used azole antifungal drugs, posing difficulties in effective treatment [9]. Immunosuppression, long-term use of broad-spectrum antibiotics and steroids, and prophylactic administration of antifungals have led to the emergence of azole resistance and a trend towards infections by NAC species. 

Species, such as *Pichia*, *Rhodotorula*, *Trichosporon,* and *Saccharomyces, *have been reported previously [10-13]. These species are capable of causing fatal infections in patients with one or more predisposing factors. Prompt identification of yeast isolates from clinical samples is imperative for the timely determination of appropriate therapy. However, this task is complicated as the conventional methods are inconclusive. The non-inclusion of the emerging pathogenic fungal species in the databases of the commercially available conventional identification kits results in misidentification [14-17]. 

Molecular-based identification tests/sequencing facilitate the accurate identification of the new species. Amplification with universal fungal primers, followed by the sequencing of the amplicon, allows for the detection of a variety of fungi in clinical specimens. The identification of fungal species is performed by sequencing the ITS regions. The ITS region is located between the highly conserved genes coding for 18S, 5.8S, and 28S rRNAs. The ITS regions are more promising for species discrimination because of its higher variability [18]. The ITS1-ITS4 primer pair is used to amplify the intervening 5.8 ribosomal DNA (rDNA) and the adjacent ITS2 and ITS3 regions. The ITS sequencing is a reliable and accurate alternative to the conventional identification methods. With this background in mind, the present study was conducted to identify the *Candida *species in HIV-infected patients by both conventional and molecular methods and also study the antifungal susceptibility pattern of the isolates.

## Materials and Methods

The study was conducted at Tagore Medical College and Hospital and Government Hospital of Thoracic Medicine in Chennai, India during 18 months (i.e., May 2016-December 2017) after ethical clearance (IEC24/March 2016). A total of 80 clinical specimens, including pus, sputum, blood, swabs, body fluids, nail, and skin scrapings, were collected from the patients suspected of candidiasis. The patients had comorbid conditions, such as tuberculosis, diabetes, tuberculous meningitis, cryptococcal meningitis, *Pneumocystis carinii* pneumonia, and fungemia, apart from HIV. In line with the research ethics, strict confidentiality was maintained throughout the study.

Samples with pus cells and budding yeast cells in direct smear, along with pseudohyphae in Gram stain or potassium hydroxide (KOH) mount, were included in the study. Growth of *Candida *species on Sabouraud dextrose agar (SDA) on two consecutive occasions was observed in the urine and sputum samples to exclude colonization. Patients receiving antifungal therapy a month prior to the study were excluded from the study.

The samples were inoculated on SDA (HiMedia-M063). In the next stage, the colonies were subjected to species identification using conventional identification methods, namely germ tube test at 37°C, chlamydospore formation, sugar assimilation tests, urease test at 25°C, ability to grow at 45°C, and CHROM agar (HiMedia, India). The isolates were also subjected to API 20C sugar assimilation test and Vitek 2 system (Vitek 2 ID-YST, BioMérieux, USA). Furthermore, the Microbial Type Culture Collection (MTCC) strains of *C. albicans* (MTCC 3017), *C. glabrata* (MTCC 3019), *C. krusei* (MTCC 9215), and *C. tropicalis* (MTCC 3421) were included as quality controls. 

The rDNA sequencing of the fungal isolates was also used to confirm the fungal species. For DNA extraction, the isolates were grown on SDA at 37°C for 24 h, and then inoculated onto yeast peptone dextrose broth and incubated for 18-24 h. Total genomic DNA of the isolates was extracted using the HiPura yeast DNA kit according to the manufacturer’s instructions. DNA concentrations and DNA/RNA ratio were measured by means of BioPhotometer D30 (Eppendorf). Polymerase chain reaction (PCR) was carried out using fungal primers for ITS1 (5′-TCCGTAGGTGAACCTGCGG-3′) and ITS4 (5′-TCCTCCGCTTATTGATATGC -3′) (Eurofins India Pvt Ltd), amplifying the ITS region of the ribosomal subunit [19]. 

The PCR product size was analyzed preliminarily by agarose gel electrophoresis (1.5% concentration), followed by visualization and analysis in Gel Doc XR+ (Bio-Rad). The purified amplified products were sequenced with an ABI 3730 XL analyzer (Applied Biosystems, Foster City, CA, USA) using standard protocols (Big Dye Terminator v3.1 cycle sequencing Kit- Applied Biosystems) and previously designed primers. Sequences were compared in the nucleotide database using the BLASTn tool (NCBI Gen Bank) for species identification. 

The antifungal susceptibility testing was performed for fluconazole (25 µg; Oxoid ^TM^, India), voriconazole (1 µg; Oxoid ^TM^, India), caspofungin (e-strip; HiMedia EM119, India), fluconazole (e-strip; HiMedia EM072, India), voriconazole (e-strip), and amphotericin B (Sigma-Aldrich) powder. The sensitivity test was performed according to the Clinical Laboratory Standards Institute (CLSI) M44-A reference document for the antifungal disk diffusion test of yeasts [20].

Sensitivity to fluconazole, voriconazole, and caspofungin was determined by disk diffusion assays in modified Mueller-Hinton agar with 2% glucose and methylene blue (0.5 µg/ml). Lawn culture was prepared from 24 h culture of SDA adjusted visually to match 0.5 MacFarland’s standard. Sterile dried Mueller-Hinton, in addition to glucose methylene blue agar plate, was used to carry out the procedure. The plates were examined for the zone of inhibition after 20-24 h. In case of insufficient growth, it was read again after 48 h of incubation. The zone diameter was measured at the point at which there was a prominent reduction in growth. 

The minimum inhibitory concentrations (MICs) of fluconazole, voriconazole, and caspofungin were evaluated by e-strips. Susceptibility pattern against amphotericin B was tested by broth dilution method in Roswell Park Memorial Institute (RPMI) 1640 broth (with glutamine and phenol red indicator without bicarbonate). The CLSI document M27-A3 reference method was used for broth dilution antifungal susceptibility testing of the yeasts [21]. 

The concentration of the tested drugs had a range of 0.0313-16 µg/ml. A suspension was made at a 1:100 dilution, followed by a 1:20 dilution of stock suspension with RPMI 1640 broth medium. Subsequently, 100 µl of inoculum was added to each well (excluding the drug control). The microtiter plates were incubated at 35°C, and the results were read after 24 h. The first well without a visible growth was taken as MIC. The susceptibility tests were performed in triplicates, and the MTCC strains were used as quality controls.

Statistical analysis (Chi-square test) was performed in SPSS software (version 20.0). Furthermore, the confidence interval was set at 95%.

## Results

The patients were clinically presented with more than one comorbid conditions apart from weight loss. The most common conditions were oral ulcers, loss of appetite, and recurrent diarrhea. The AIDS-defining illnesses, such as tuberculosis, were detected in 52.5% of the patients, followed by tuberculous meningitis (0.06%), cryptococcal meningitis (0.04%), *Pneumocystis carinii *pneumonia (0.05%), and recurrent herpes zoster (1.3%). 

***Prevalence of candidiasis***

The prevalence rate of candidiasis was obtained as 75% (n=60) in the HIV-infected patients. All isolates were identified by both phenotypic and molecular methods. In this regard, *Candida tropicalis* was the predominant isolated species with a prevalence rate of 30%. Table 1 presents the distribution of the identified species. In the present study, the rarely emerging species were isolated as etiological agents. *Kodamaea ohmeri, C. orthopsilosis, *and *Hanseniaspora opuntiae* were isolated from the blood*, *nail, and pus samples, respectively. *Candida tropicalis* was the predominant species in all samples. Based on the results, the sample type did not show a specific pattern of *Candida* species (*P>0.05*).

In the SDA, *K. ohmeri* colonies were smooth and pasty; furthermore, they were wrinkled with further incubation. On CHROMagar *Candida* medium (HiMedia), the color of *K. ohmeri* colony changed from pink to blue in a period of 48 h as reported earlier [22]. On the SDA, after 24-48 h of incubation, *H. opuntiae* colonies were cream-colored with butyrous consistency, which were slightly raised at the center with an entire to slightly undulated margin. 

On the cornmeal agar, pseudomycelium was formed after 48-72 h, and only glucose was assimilated. In this regard, the other carbohydrates used (i.e., galactose, xylose, sucrose, maltose, trehalose, and lactose) were not assimilated. Based upon phenotypic characteristics, the species cannot be identified. However, these species were confirmed by the molecular method.

Based on our results, candidiasis had a higher prevalence rate in male patients (63.3%), compared to that in the female patients (46.7%). Nonetheless, the difference was not statistically significant (P>0.05). The age group of 31-40 years was predominantly infected with a prevalence rate of 28%. Although the patients within the age group of 31-40 years had the highest prevalence of infection, the results of Chi-square analysis showed no significant association between the prevalence of candidiasis and age (*P>0.05*). 

***Antifungal susceptibility test***

According to the results, 63.3% and 18.3% of the *Candida* isolates were resistant to fluconazole and voriconazole, respectively. The sensitivity pattern differed across various isolates. In this regard, fluconazole MICs were higher for *C. glabrata* and *C. tropicalis*, compared to those for others. The MIC value for the fluconazole-resistant strains ranged from > 64 to > 256 µg/ml. One of the *C. albicans* strains was resistant to amphotericin B; in addition, two NAC isolates were resistant to caspofungin. 

The MICs of amphotericin B and caspofungin had the ranges of 0.25-1 and 0.012-2 µg/ml, respectively. *Kodamaea ohmeri* isolate was found to be resistance to fluconazole and voriconazole and susceptible to caspofungin. This isolate also showed an increased amphotericin B MIC value (1 µg/ml). The fluconazole and caspofungin MICs for *K. ohmeri* strain were obtained as > 256 and 0.12 µg/ml, respectively*. *

**Table 1:** Prevalence of candidiasis in various immunosuppressed conditions

**Conditions**	***Candida*** ** species**							**Growth**	**No growth**	**Total**
	***C. albicans***	***C. tropicalis***	***C. krusei***	***C. kefyr***	***C. glabrata***	***C. parapsilosis***	**Others**			
HIV	3	5	1	0	0	2	0	11 (52.4%)	10 (47.6%)	21
HIV and tuberculosis	4	5	4	1	4	4	1	23 (82.1%)	5 (17.9%)	28
HIV, tuberculosis, and diabetes	4	4	1	0	0	0	0	9 (64.3%)	5 (35.7%)	14
HIV, tuberculous, and meningitis	1	2	1	0	0	0	1	5 (100%)	0	5
HIV and cryptococcal meningitis	1	1	0	0	0	0	1	3 (100%)	0	3
HIV and pneumocystis carinii	2	0	0	0	1	1	0	4 (100%)	0	4
HIV, tuberculosis, and fungemia	2	1	1	0	0	0	1	5 (100%)	0	5
**Combined Data**										
HIV	*C. albicans*		NAC species					Total		
	17 (28.3%)		43 (71.7%)					60		
*P>0. 05*										

NAC: non-*albicans Candida*

**Table 2:** Antifungal susceptibility pattern of Candida species

***Candida species***	**Fluconazole**			**Voriconazole**				**Amphotericin B**				**Caspofungin**	
	**S**	**SDD**	**R**	**S**	**SDD**		**R**	**S**	**SDD**	**R**	**S**	**SDD**	**R**
*C. albicans*	5	2	10	10	2		5	16	0	1	15	1	1
*C. tropicalis*	1	5	12	12	3		3	17	1	0	17	0	1
*C. krusei*	0	0	9	5	2		2	8	1	0	8	1	0
*C. glabrata*	3	1	1	5	0		0	5	0	0	5	0	0
*C. kefyr*	0	0	1	1	0		0	1	0	0	1	0	0
*C. parapsilosis*	1	2	3	5			0	6	0	0	6	0	0
*C. dubliniensis*	0	0	1	1	0		0	1	0	0	1	0	0
*C. orthopsilosis*	0	0	1	1	0		0	1	0	0	1	0	0
*K. ohmeri*	0	0	1	0	0		1	1	0	0	1	0	0
*H. opuntiae*	0	0	1	1	0		0	-	-	-	1	0	0
**Combined Data**													
*C. albicans*	5	2	10	10		2	5	16	0	1	15	1	1
NAC species	5	8	30	31		6	6	40	2	0	41	1	1
*P-value*	>0.05			>0.05				>0.05			>0.05		

NAC: non-*albicans Candida*

*Hanseniaspora* *opuntiae* strain was sensitive to all the antifungals investigated in the present study, except fluconazole. The MIC value for fluconazole was obtained as 128 µg/ml. The susceptibility pattern to amphotericin B could not be determined since the isolate did not grow well on the RPMI 1640 medium. Table 2 demonstrates the antifungal sensitivity pattern of the *Candida* isolates.

***Nucleotide Sequence Accession Number***

The sequences of *K. ohmeri *and *H. opuntiae* isolates were deposited in the NCBI GenBank database with the accession numbers of MF196235 and MF196236, respectively.

## Discussion

In the current study, candidiasis had an prevalence rate of 75%. A similar prevalence rate was noted in the earlier studies performed by Pruthvi *et al.,* Nagalingeswaran *et al.,* Singh *et al.,* and Anupriya Wadhwa *et al.* reporting the prevalence rates of 71%, 70%, 65%, and 50% in HIV-positive patients, respectively [23-26]. In line with the results of an earlier study carried out by Picardi *et al.* [27], the findings of the present study noted a shift towards NAC species. In the mentioned study conducted in the USA during 2004-2009, NAC strains were more frequently isolated from neutropenic patients. Indian studies also reported an increase in the prevalence of NAC species [28-30]. 

In this study, the patients were within a wide age group of 12-65 years. Candidiasis was more common among the patients with an age range of 31-40 years. The studies performed by Vajpayee *et al.* (2003) and Ravinder *et al.* (2016) also recorded similar observations [31, 32]. The results of the current study also showed that candidiasis had a male preponderance (63.3%). The higher prevalence of this infection in the male population is due to HIV infection resulting from unprotected sex with sex workers due to job-related trips that lead to separation from the spouse for a long time [32].

The current study led to the identification of rarely emerging species. In the present research, the conventional methods failed to identify certain fungal species. The morphological identification by Gram staining initially led to the suspicion of *Candida* species. While the conventional methods were inconclusive, the molecular method facilitated the identification process. 

In 1984, *K. ohmeri* was isolated from a pleural fluid; nonetheless, at that time, it was considered as a contaminant [33]. Since then, this species has been considered a true clinical pathogen, especially in patients with underlying immunosuppression in the case reports mostly conducted in Asia (e.g., Korea, Turkey, and India) [34]. There are also reports from North America and Brazil in this regard [35, 36].

Chakrabarti *et al.* conducted a study including the largest pediatric population among neonates in intensive care units (ICUs) from a tertiary care hospital in North India [37]. In the mentioned study, the major risk factors were reported as prematurity, low birth weight, prolonged ICU stay, use of medical devices, prosthetic valves, use of broad-spectrum antibiotics, total parenteral nutrition, immunosuppression (e.g., leukemias and lymphomas), neutropenia, diabetes, and chronic renal failures. They observed only one case of infection in a previously healthy child with no predisposing conditions, except for encephalitis. Accurate identification is a matter of paramount importance, especially with regard to the fact that *K. ohmeri* has the potential to cause hospital outbreaks.

The API 20C for carbohydrate fermentation is the gold standard for the identification of yeasts. In this panel of sugars, *K. ohmeri *assimilates raffinose rather than D-xylose, whereas *C. tropicalis* can assimilate D-xylose rather than raffinose [38, 39]. However, this diagnostic method can misidentify some species, like *C. haemulonii* or *C. parapsilosis, *as *K. ohmeri.* However, the majority of the laboratories in India do not perform entire sugar assimilation for *Candida* species. The VITEK 2 ID-YST system facilitates more accurate identification, although there have been some misidentifications with *C. haemulonii *[38].

Dominguez *et al.* (2012) reported that MALDI-TOF resulted in the identification of *K. ohmeri* with a higher accuracy than API20C; however, it had a comparable accuracy to that of PCR [40]. These isolates had been misidentified as *C. haemulonii* by the API20C method. Nonetheless, in Indian scenario, molecular identification is seldom performed for cost-effectiveness. The ITS region is an essential and effective tool for differentiating the rare species that are frequently misdiagnosed, bearing in mind that reliable sequence databases should be used [38]. 

*Kodamaea ohmeri* strain isolated in this study was resistant to fluconazole and voriconazole. No intrinsic resistance of *K. ohmeri* to antifungals has been reported yet. This strain is frequently resistant to fluconazole; therefore, it should not be used for empirical therapy [41]. There are a number of reports regarding echinocandin resistance. In line with a study carried out by Chakrabarti *et al. *[37], the present study demonstrated an increased amphotericin B MIC value for this species. Therefore, antifungal treatment should be adjusted according to the susceptibility reports of the clinical isolates. The CLSI has defined that fungal susceptibility breakpoints for this yeast are the same as those for *C. albicans*.

In the present study, one of the detected isolates was *H. opuntiae*, which was isolated from the pus samples obtained from a female patient with other comorbid conditions like tuberculosis and diabetes. This is the first time that this species has been reported at this geographical area. Literature is indicative of the isolation of three *H. opuntiae *strains from a Tunisian hospital [42]. There are limited reports about the etiology of this agent. 

*Hanseniaspora* *opuntiae *is not still considered as an emerging fungal pathogen as it has not been isolated from any disease conditions in the previous studies, except for one study. However, in the present study, the fluconazole-resistant *H. opuntiae* was isolated*. *The resistance pattern of this species heightens the suspect of the presence of this strain in our geographical area for a long period of time. An extensive epidemiological study is required to confirm these results and figure out whether this fungus can be considered an emerging fungal pathogen, especially in the immunocompromised patients. 

In this study, resistance to fluconazole was higher than that to other investigated antifungals. Many studies have estimated the prevalence of fluconazole resistance as 6-36%, 15%, and 5-10% [43]. This may be due to the long-term prophylactic usage of fluconazole, immune status (low CD4 count), presence of other comorbid conditions, and increased prevalence of NAC species [44].

## Conclusion

In the present study, the emerging yeast species, such as *K. ohmeri, C. orthopsilosis,* and *H.* *opuntiae,* were identified by molecular techniques as the phenotypical identifications were inconclusive. The study underscores the need for the prompt and timely identification of clinical yeast isolates because many rare species are emerging and capable of causing life-threatening infections and outbreaks. 

In laboratories where molecular diagnostic methods are not available, the alternative services of reference laboratories can be utilized as cost-effective measures. As the findings indicated, the antifungal drug resistance is increasing, and the emerging species are multidrug-resistant. Consequently, antifungal susceptibility testing should be made mandatory for effective patient management. 
